# Utilization of urethral plate in hypospadias surgery

**DOI:** 10.4103/0970-1591.40615

**Published:** 2008

**Authors:** Warren T. Snodgrass

**Affiliations:** Department of Urology, Pediatric Urology Section, Children's Medical Center and the University of Texas Southwestern Medical Center, 2350 Stemmons Freeway Suite F4300 Dallas, Texas 75207, USA

**Keywords:** Hypospadias, surgery, urethral plate, tubularized incised plate

## Abstract

**Purpose:**

Recognition the urethral plate comprises tissues that normally should have created the urethra was an important milestone in hypospadias surgery, giving rise to new operative repairs - most notably the tubularized incised plate technique. This article reviews the current state of the art for hypospadias repair using tubularized, incised plate (TIP).

**Materials and Methods:**

Personal experience and literature reports were reviewed to summarize use of TIP urethroplasty for distal, proximal, and re-operative hypospadias repairs.

**Results:**

The TIP can be used to correct all distal and most proximal hypospadias. The major contraindication is ventral curvature that leads to urethral plate transection for straightening, which is only necessary in some proximal cases. Reoperations can also be performed using TIP provided the urethral plate has been maintained and is not grossly scarred. Complication rates are comparable to previously used techniques, while cosmetic appearance after TIP is considered superior to other available procedures.

**Conclusions:**

Recognition of the urethral plate and its incorporation into the neourethra has revolutionized hypospadias repair. The most commonly used operative procedure today is TIP.

## INTRODUCTION

The most important development in modern hypospadiology is recognition the urethral plate comprises tissues distinct from glans and penile skin that normally should have formed the urethra. This plate consists of surface epithelium overlying well-vascularized connective tissue which can be preserved for urethroplasty, rather than fibrous “chordee” bands previously thought to cause ventral curvature. Furthermore, midline sagital incision of the urethral plate to widen it heals without clinical scarring, enabling hypospadias repair without supplemental skin flaps.

These observations established the foundation of current hypospadias surgery in which there is increased reliance on tubularization of the urethral plate for urethroplasty or creation of a neourethral plate for tubularization when the native plate has been excised or is unsuitable for repair. When skin flap repairs are performed, today they often incorporate the urethral plate.

This review considers the state of the art in hypospadias surgery emphasizing the role of the urethral plate.

## TIP REPAIR

**Figure 1:**
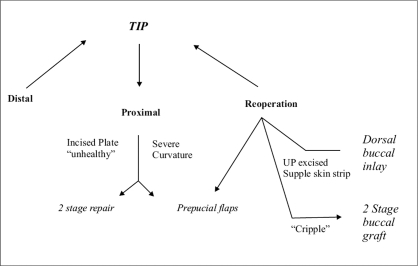
Algorithm for current hypospadias surgery

Despite recognition of the urethral plate, simple tubularization was not thought possible in the majority of cases because the neourethra would be too narrow unless the plate was already quite wide and/or deeply grooved. Midline incision was subsequently found to widen the plate for tubularization into a neourethra of satisfactory diameter and to heal without apparent scar contracture. This observation became the basis of Tubularized, Incised Plate (TIP) urethroplasty,[1] which today is the most popular hypospadias repair performed worldwide. The resultant shift away from skin flap repairs[2] is apparent in the algorithm for current hypospadias surgery shown in Figure 1.

## DISTAL

**Figure 2:**
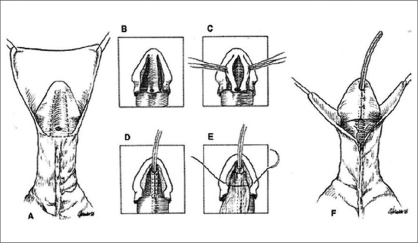
Steps of TIP

The technique for TIP repair is well-described and illustrated in Figure 2. Key points include deep incision of the urethral plate to near the underlying corpora cavernosa and taking care during tubularization not to suture too far distally and thereby create meatal stenosis. I have suggested tubularization should not extend beyond the midportion of the glans wings, which is about 3 mm from the tip of the plate. The neomeatus should be oval, not rounded, as in a normal child.

The complication rate in my last 120 patients undergoing distal TIP is 2.5%, comprising two fistulas and one glans dehiscence. A recent review of the world's literature from 1994 to 2004 found overall complications in 9% (0-21%) shown in Table 1. As with all hypospadias procedures, the most common problem was fistulas, but it is noteworthy that meatal stenosis was uncommon and neourethral strictures rare.

**Table 1:** Tubularized, incised plate outcomes for distal hypospadias

Authors	Year	Number of patients	Mean follow-up (Months)	Points total comp	Fistula	Meatal stenosis	Dehiscence	Stricture
Snodgrass	1994	16	22	0	-	-	-	-
Snodgrass	1996	129 (eight reoperations)	NS	10	5	3	2	-
Steckler, Zaontz	1997	31	3	0	-	-	-	-
Ross, Kay	1997	15	< 12	0	-	-	-	-
Elbakry	1999	21	20	4	4	4	-	-
Sugarman *et al.*	1999	25	10	1	1	-	-	-
Oswald *et al.*	2000	30	15	1	-	-	1	-
Holland *et al.*	2000	60	27	9	6	3	-	-
Dayanc *et al.*	2000	20	20	2	1	1	-	-
Guralnick *et al.*	2000	28	9	8	6	2	-	-
Borer *et al.*	2001	156	6-38	7	6	1	-	-
Smith	2001	53	1	0	-	-	-	-
Cheng *et al.*	2002	414	4-66	1	0	1	-	-
El-Sherbig	2003	64	6	9	7	2	-	-
Jayanthi	2003	110	9	1	1	-	-	-
Samuel, Wilcox	2003	65	4	4	3	-	1	-
Leclair *et al.*	2004	1-62 (6 midshaft)	12	13	9	4	-	-
Elicevik *et al.*	2004	324	6-60	75	39	32	12	3
Lorenz *et al.*	2004	22	3-6	1	1	1	-	-
Total		1745		146 (8%)	89 (5%)	54 (3%)	16 (1%)	

NS-not stated

I am not aware of any contraindication to TIP for distal repair and have not used any alternative technique in the past 10 years. Unlike older procedures that were designed for specific anatomic variations, TIP is useful regardless of whether the urethral plate is flat or deeply grooved, narrow or wide before incision.[3]

Popularity of TIP also relates to its cosmetic outcomes, as the operation reliably creates a vertical, slit neomeatus. In an attempt to objectively compare TIP to other techniques, postoperative standardized photographs after TIP, Mathieu and onlay flap repairs were judged by a panel of health care workers blinded to the operation.[4] This report found appearance of the penis after TIP significantly superior to skin flap urethroplasty. To test claims the operation creates a normal appearing penis, we recently completed a questionnaire survey of parents after TIP repair, comparing their responses to parents whose boys were normal and underwent circumcision.[5] There were no differences in opinion regarding penile appearance between the two groups, as families of boys with hypospadias surgery thought the results looked normal.

Versatility allowing use for all distal repairs, a straightforward surgical technique, low-complication rates, and superior cosmetic outcomes account for popularity of TIP urethroplasty. A recent survey of hypospadiologists throughout the Americas and Europe found 92% preferred TIP for distal hypospadias surgery.[2]

## MIDSHAFT

Midshaft hypospadias defects typically have been reported combined with either more distal or proximal repairs. However, my complication rate of 13% for these cases vs. 3% for distal and 25% for proximal TIP demonstrates outcomes are significantly different depending upon the extent of the lesion.[6]

We recently reported experience with 35 consecutive midshaft repairs, finding TIP successful in each case.[6] As with more distal surgeries, I am not aware of a contraindication to this procedure for midshaft hypospadias. Others are increasingly using TIP for midshaft defects, with the survey mentioned above reporting 83% used this technique, while the second most common technique was onlay flap.

## PROXIMAL

In a recent review of 56 proximal shaft to perineal hypospadias cases, we used TIP in 36 (64%).[6] The remaining 20 patients had either an unhealthy appearing incised plate[1] or significant ventral curvature leading to plate transection. I previously reported the occasional finding of an incised plate that grossly seems deficient of subepithelial connective tissues.[7] However, the most common contraindication to TIP has been curvature persisting after the penis is degloved and ventral dartos tissues dissected. Following the suggestion of Castagnola and Mollard,[8] I have elevated the corpus spongiosum or the entire urethral plate from the surface of the corpora cavernosa before determining bending justified transecting the plate.

Historically, hypospadias surgery emphasized proximal repairs which are most likely to have significant curvature. The traditional explanation for bending was fibrous bands under the penile skin that required excision for straightening. Others disputed this interpretation arguing the penis is normally curved during initial formation and that the arrest in development producing hypospadias occurs during this period.[9] Some noted inability to identify so-called chordee bands during surgery and recent reports note the plate is comprised of well-vascularized tissues, not fibrous bands.[10,11] Most important were the observations that curvature often is minimally improved by transecting the plate and dissecting ventral “;chordee” tissues.[12]

Today, it is more widely accepted that ventral curvature may be associated with shortened ventral shaft skin, dartos, corpus spongiosum, urethral plate, and/or corpora cavernosa. Accordingly, after extensive dissection of these tissues, curvature that persists is corrected by either dorsal plication or ventral corporal incision and grafting, depending upon its extent.

Options for urethroplasty after the plate is transected include one stage-tubularized prepucial flaps or various staged repairs, using either flaps or grafts. Dissatisfaction with these options has led to reconsideration of means to straighten the penis to determine if the urethral plate can be maintained despite curvature >30°. Previously Turner Warwick reported bulbar urethral mobilization as an adjunct during hypospadias surgery[13] and stricture urethroplasty long has involved extensive mobilization of the urethra. Bhat *et al.*[14] found dissection of the urethral plate and more proximal bulbal urethra together off the corpora cavernosa allowed straightening without need to transect the plate, allowing TIP. During the past year, I too have found the urethra and urethral plate can be mobilized to gain access to the ventral surface of the corpora, where multiple superficial incisions into the tunica albuginea were made for straightening. Then the urethra was advanced distally and sutured back to the corpora and the plate incised and tubularized.

There were six publications regarding proximal TIP between 1998 and 2003, with a complication rate of 14% (4-33%) [Table 2]. However, these combined midshaft and more proximal repair outcomes and none described decision making between TIP and other options for more proximal operations. Therefore, selection bias may have influenced results. Nevertheless, the survey by Cook *et al.*[6] found 43% of respondents used TIP for proximal repairs when there is no ventral curvature, the same percentage that preferred onlay prepucial flaps.

**Table 2:** Prior reports of midshaft to proximal tubularized, incised plate

Authors	Date	Patients	Ventral curvature (%)	Mean follow up months	Total patient complications (%)	Complications				
						
						Fistulas	Meatal stenosis	Neourethral stricture	Dehiscence	Recurrent curvature
Snodgrass *et al.* (multicenter)	1998	16 midshaft	11 (69)	NS	3(11)	1	1	0	1 complete	NS
		11 proximal	10 (91)							
Chen *et al.*	2000	10 midshaft	9 (23)	12.5	2(20)	2*	1*	0	NS	NS
		27 proximal			5(19)	4*	3*	0	NS	NS
Borer *et al.*	2001	16 midshaft	NS	6-38	1(6)	1	NS	NS	NS	NS
		9 proximal			2(22)	2	NS	NS	NS	NS
Snodgrass and Lorenzo	2002	13 midshaft	5 (38)	9	2(15)	1	0	1	0	0
		20 proximal	13 (65)		9(45)	6*	1*	0	1	2
Cheng *et al.* (multicenter)	2002	100 “midshaft to penoscrotal“	NS	4-66	4(4)	3	1	0	NS	NS
Samuels and wilcox	2003	18 proximal	4 (22)	4	4(22)	1	0	0	3 glans	NS
Mustafa	2005	1 midshaft	1 (100)	NS	4(31)	3	1	0	NS	NS
		12 proximal	1 (8)							
Total		253			36(14%)				

NS - not stated.

* One patient with more than 1 complication

Today the potential role of TIP in proximal hypospadias remains incompletely defined. However, the same can be said for onlay flaps as there are only three reports using this technique specifically for proximal hypospadias [Table 3]. Similarly, publications concerning tubularized prepucial flaps often have combined distal and proximal hypospadias repairs together, making it more difficult to determine outcomes for more severe cases. It appears complication rates in the range 35-50% are expected with proximal tubularized flaps.

**Table 3:** Onlay prepucial flap proximal hypospadias

Authors	Date	Patients	Ventral curvature (%)	Mean follow up months	Total patient complications (%)	Complications						
						
						Fistuals	Meatal stenosis	Neourethral stricture	Dehiscence	Recurrent curvature	Diverticulum	Other
Mollard *et al.*	1991	22 mid to proximal shaft	22 (100)	NS	0	0	0	0	0	0	0	
Barrosco *et al.*	2000	12 midshaft	29 (62)	15.2	12 (21)	2*	2*	NS	2*	2*	4*	
		35 proximal				6						
Samuel *et al.*	2001	17 proximal	10 (59)	3.2 years	10(59)†	10	2		0			2 BXO

NS - not stated.

* One patient with more than 1 complication.

† Unclear in report if patients with MS or BXO also had fistuals, or were not counted as having complications

Despite the longer history of flap hypospadias repair, there are few data regarding outcomes in adults operated as children. However, one such study[16] used questionnaires to determine voiding and sexual function in 27 teens and adults a mean of 13 years after surgery using a staged tubularized prepucial flap technique. Approximately half originally presented with midshaft hypospadias, while the remainder had more proximal defects. Of these, 10 reported “minor spraying” of their stream and 10 milked urine after voiding. Similarly, of those who had experienced ejaculation, 43% manually expressed semen from the urethra. The authors concluded it is not surprising there might be stasis in long neourethras constructed with skin even in the absence of diverticulum.

## REOPERATIONS

Boys presenting with persistent hypospadias after prior attempts at correction may still have the urethral plate intact. If the plate has been conserved and it appears to be supple without gross scarring, incision, and tubularization can be considered for reoperation. In my experience, most patients undergoing TIP for reoperation previously failed only one or in a few cases two, previous operations and the urethral plate seemed little altered from the un-operated state.[16] Results from the published series are shown in Table 4 and it is important to emphasize the observation that fistulas were more common in TIP reoperations when local dartos tissues were just approximated over the neourethra in contrast to when a flap of dartos was fashioned to cover the suture line.

**Table 4:** Tubularized, incised plate reoperations

Authors	Numbers of patients	Mean number of prior operations (range)	Complication rate (%)	Fistulas (%)	Meatal stenosis (%)	Urethral STX (%)	Diverticulum	Dehiscence
Shamberg *et al.*	13	2.5(1-6)	15	8*	8	0	0	8%
Borer *et al.*	25	Not stated	24	20	4	0	0	0
Yang *et al.*	25	2.5	Not stated	28	52	8	Not stated	Not stated
Snodgrass and Lorenzo	15	1(1-2)	20	13	0	0	0	6%

* One patient had both glans dehiscence and a fistula

Reoperations when the plate has been excised or scarred have been influenced by modern concepts regarding the urethral plate. Although beyond the scope of this review, both inlay grafting [17,18] and staged grafting procedures [19,20] create a neourethral plate for tubularization.

## CONCLUSIONS

Recognition and use of the urethral plate has revolutionized hypospadias surgery during the past decade. Currently TIP repair is most widely used for distal and midshaft hypospadias, but while early outcomes data are promising there are, to date, no reports regarding its long-term results. Proximal hypospadias remains an area of controversy, as it is not yet clear whether TIP or flap repairs should be preferred for more severe defects. Reoperations can also be performed using TIP when the urethral plate remains supple. The major contraindication to TIP presently is ventral curvature leading to plate transaction during straightening, but recent experience with mobilization of the urethral plate and urethra proximally to near the sphincter suggests the plate often can be maintained while correcting bending.
